# Synthesis of Multi-Substituted Pyrrole Derivatives Through [3+2] Cycloaddition with Tosylmethyl Isocyanides (TosMICs) and Electron-Deficient Compounds

**DOI:** 10.3390/molecules23102666

**Published:** 2018-10-17

**Authors:** Zhengning Ma, Zicheng Ma, Dawei Zhang

**Affiliations:** 1 College of Chemistry, Jilin University, Changchun 130012, China; mazn8215@mails.jlu.edu.cn (Z.M.); mazc8215@mails.jlu.edu.cn (Z.M.); 2 College of Plant Science, Jilin University, Changchun 130062, China

**Keywords:** pyrrole heterocycles, TosMICs, electron-deficient compounds, Van Leusen pyrrole synthesis, [3+2] cycloaddition

## Abstract

Pyrrole and its polysubstituted derivatives are important five-membered heterocyclic compounds, which exist alone or as a core framework in many pharmaceutical and natural product structures, some of which have good biological activities. The Van Leusen [3+2] cycloaddition reaction based on tosylmethyl isocyanides (TosMICs) and electron-deficient compounds as a substrate, which has been continuously developed due to its advantages such as operationally simple, easily available starting materials, and broadly range of substrates, is one of the most convenient methods to synthetize pyrrole heterocycles. In this review, we discuss the different types of two carbon synthons in the Van Leusen pyrrole reaction and give a summary of the progress of these synthesis methods in the past two decades.

## 1. Introduction

Pyrrole and its polysubstituted derivatives are important five-membered heterocyclic compounds, which exist alone or as a core skeleton in many pharmaceutical and natural product structures, some of them have good bioactivity such as antibacterial [1,2], antifungal [3,4], anti-inflammatory [5,6], antiviral [7], antimalarial [8], anticancer [9,10], antiparasitic [11], etc., and can also be used as enzyme inhibitor in the organism [12,13]. Some typical pyrrole derivative chemical structures and the physiological functions are summarized in the following Table 1.

**Scheme 1:**
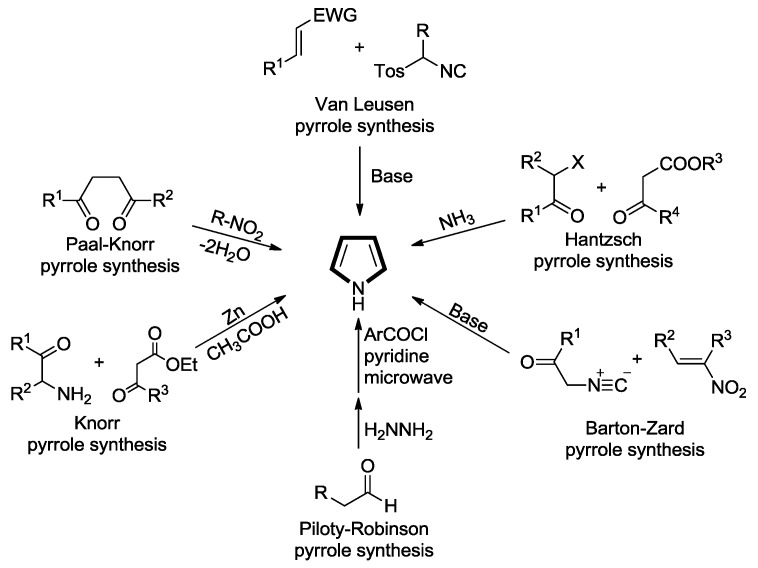
Typical cycloaddition methods for pyrrole heterocycle and its derivatives.

Since pyrrole and its multi-substituted derivatives play an important role in organic synthesis as well as in biology, syntheses of five-membered heterocyclic pyrrole compounds have always been valued by researchers. Over the last decades, there are many methods for synthesizing pyrrole compounds in laboratory routes [14], and the classical methods include Knorr pyrrole synthesis [15], Paal-Knorr pyrrole synthesis [16], Hantzsch pyrrole synthesis [17], Barton-Zard reaction [18], Van Leusen pyrrole synthesis [19], and Piloty–Robinson pyrrole synthesis [20]. These synthesis methods are summarized in Scheme 1.

As shown in Scheme 1, the method for synthesizing pyrrole based on the [3+2] cycloaddition reaction of TosMIC as 3-atom synthon with electron-deficient olefins is also known as the Van Leusen pyrrole synthesis. It was first reported by the Van Leusen et al. in 1972. Van Leusen and co-workers used TosMICs with ester-containing double bond compounds to synthesize a series of 3,4-disubstituted pyrrole compounds under basic conditions [19]. Subsequently, they extended the substrate scope to include electron-withdrawing groups on electron-deficient olefins, such as α,β-unsaturated cyano, sulfonyl, nitro, and sulfonyl groups, which enriched the structure diversity of the resulting pyrrole compounds [21,22]. Therefore, the Van Leusen [3+2] cycloaddition reaction is one of the most convenient methods to synthetize pyrrole heterocycles, which has been continuously developed over the ensuing years due to its advantages such as operationally simple, easily available starting materials, and broad range of substrates.

**Scheme 2:**
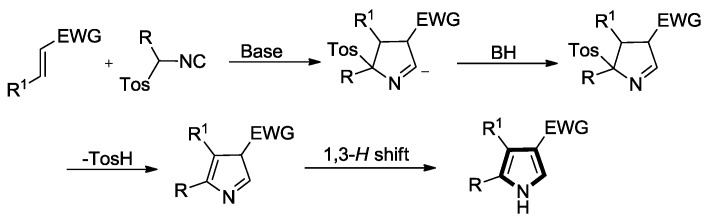
Mechanism of pyrrole compounds formed by [3+2] cycloaddition between TosMICs and electron-deficient alkenes.

TosMIC is a colorless, odorless, stable solid that can be stored at room temperature. It is an important organic synthesis intermediate, and widely used in the synthesis of five-membered nitrogen-containing heterocycles [23]. Under the Van Leusen pyrrole synthesis reaction conditions, TosMIC loses a proton to form a carbanion under the action of a base because of the electron-withdrawing effect of the sulfone and isocyanide. The carbon anion attacks on the α,β-unsaturated compound, undergoes an intramolecular [3+2] cycloaddition reaction, and causes the leaving of the tosyl group to form the final heterocyclic compound. The mechanism of [3+2] cycloaddition reaction between TosMICs and electron-deficient alkenes to form pyrrole derivatives is shown in Scheme 2.

In recent reports, it has been found that this reaction occurs selectively at the position of a less polar double bond. The use of electron-deficient compounds having an electron-withdrawing group and a relatively stable structure, or a solvation effect in the reaction system to stabilize the structure of the electron-deficient compound, can significantly increase the rate and yield of the reactions.

In this paper, we review the research progress of the synthesis of pyrrole derivatives through the [3+2] cycloaddition reaction between TosMICs and different kinds of 2-carbon synthons based on the Van Leusen pyrrole synthesis method in the past two decades.

## 2. Synthesis of Pyrrole Derivatives by [3+2] Cycloaddition of TosMICs with Alkenes

**Scheme 3:**
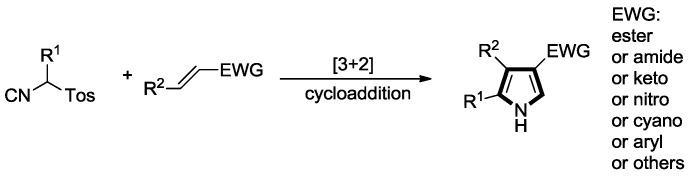
Synthetic protocols for polysubstituted pyrroles from TosMICs and electron-defect alkenes.

In 1972, Van Leusen and co-workers firstly reported that TosMICs can react with electron-deficient alkenes under basic conditions to produce 3-substituted pyrrole derivatives [19]. They found that there are [3+2] cycloadditions occurring in alkenes with different electron-withdrawing groups. As shown in Scheme 3, the electron-withdrawing groups may be esters, amides, ketones, nitros, cyanos, aryls, etc. [24,25,26,27,28,29,30,31,32,33,34,35,36,37,38,39,40,41,42,43,44,45,46,47,48,49,50,51,52,53,54,55,56,57,58,59,60]. Based on the different types of electron-withdrawing group attached to the alkenes, they are classified and described in order (Scheme 3).

### 2.1. Alkenes with an Ester Group

**Scheme 4:**
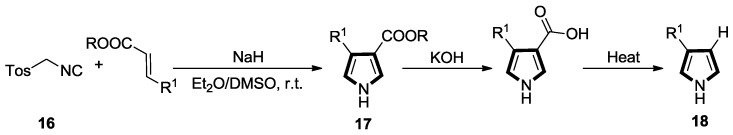
Synthesis of 3, 4-substituted and 3-disubstituted pyrrole compounds **17** and **18**.

As early as in the 1990s, Van Leusen and co-workers developed a process in which TosMIC **16** reacts with a Michael acceptor to form 3,4-disubstituted pyrrole compounds **17** or 3-substituted pyrrole compounds **18**. This procedure necessarily installs the activating Z group of the Michael acceptor at the 3-position of the pyrrole ring formed (Scheme 4) [22,24]. 

**Scheme 5:**
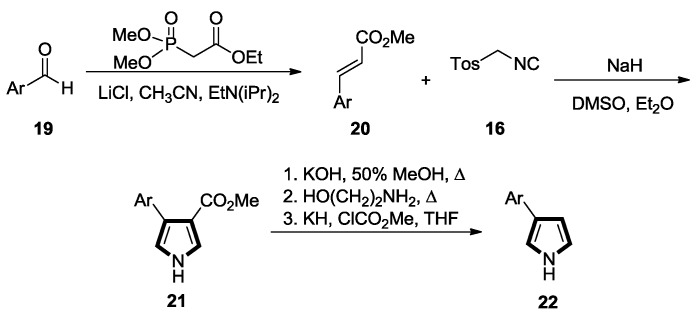
Synthesis of 3-arylpyrroles **22**.

In 1997, the Trudell group described an expeditious method for the synthesis of 3-aryl-substituted pyrroles. The 3-arylpyrroles **22** were prepared in a short reaction sequence from the readily available aryl aldehydes **19**. The aldehydes **19** were converted into the corresponding methyl 3-arylacrylate esters **20** using a Wadsworth-Emmons olefination procedure. Treatment of **20** with TosMIC **16** afforded the 4-aryl-3-(methoxycarbonyl)-pyrroles **21**. Then the ester moieties were hydrolyzed to the corresponding carboxylic acids with excess KOH in 50% MeOH. The acid derivatives were then decarboxylated by heating in 2-ethanolamine to give the 3-arylpyrroles **22** in good yield (Scheme 5) [25]. 

The electron neutral or electron-deficient aryl vinyl esters such as cinnamic acid esters could be successfully employed in the cyclization reaction. However, the TosMIC **16** addition reaction with **20** which possessed electron-rich substituents on the aryl ring did not yield the desired pyrroles, but rather gave intractable mixtures.

**Scheme 6:**
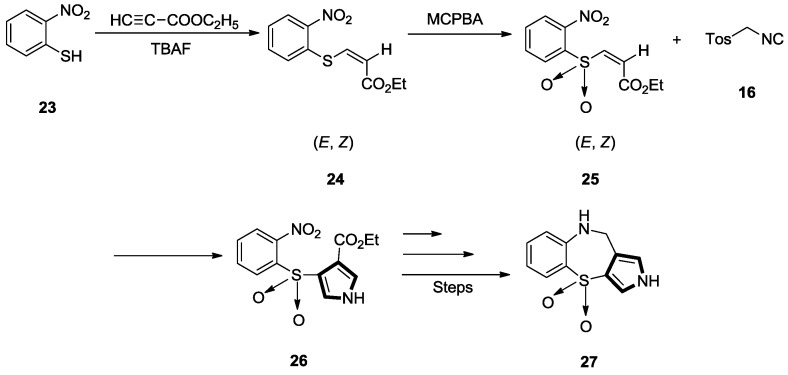
Synthesis of 2*H*-pyrrolo[3,4-*b*][1,5]pyrrolobenzothiazepine **27**.

The next year, Di Santo et al. pioneered the synthesis of 2*H*-pyrrolo[3,4-*b*][1,5]pyrrolo-benzothiazepine **27**. They started with tetrabutylammonium fluoride (TBAF)-catalyzed reaction of 2-nitrothiophenol **23** with ethyl 2-propynoate to afford ethyl 3-(2-nitrophenylthio)propenoate **24**. Then they performed a transformation of the sulfur derivative into the sulfone analogue by the use of *m*-chloroperbenzoic acid (MCPBA). Afterwards, the novel *E*/*Z* mixture **25** was reacted with TosMIC **16** to form ethyl 4-(2-nitrophenylsulfony1)-1*H*-pyrrole-3-carboxylate **26** as the sole product. 2*H*-pyrrolo[3,4-*b*][1,5]pyrrolobenzothiazepine **27** can be synthesized by using compound **26** as raw material (Scheme 6) [26].

**Scheme 7:**
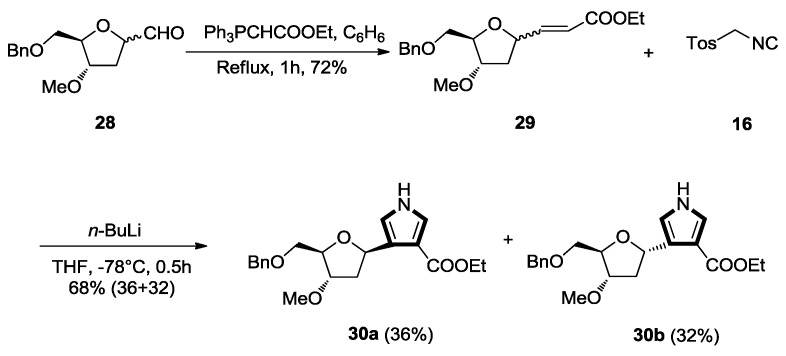
Synthesis of pyrrole *C*-nucleosides **30a** and **30b**.

In 2007, Krishna’s group found that aldehyde **28** on treatment with (ethoxycarbonylmethylene)-triphenylphosphorane in refluxing benzene was converted to the α,β-unsaturated ester **29** (72%), and treatment of **29** with potassium salt of TosMIC **16** afforded the corresponding pyrrole C-nucleosides **30a** (36%) and **30b** (32%) (Scheme 7) [27].

**Scheme 8:**
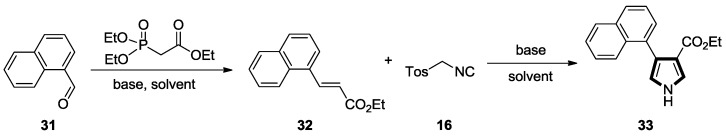
Synthesis of ethyl 4-substituted-1*H*-pyrrole-3-carboxylate **33.**

In 2008, Shin’s group developed a synthesis of ethyl 4-substituted-1*H*-pyrrole-3-carboxylates **33** from aldehyde **31**, in which they synthesized α,β-unsaturated ester **32** from aromatic or aliphatic aldehydes by the Horner-Wadsworth-Emmons reaction and subsequently reacted it with TosMIC **16** in the presence of sodium *t*-amylate in toluene. In this reaction, the solvent, toluene, can be used in both reaction and crystallization, which makes it more practical and greener (Scheme 8) [28]. 

**Scheme 9:**
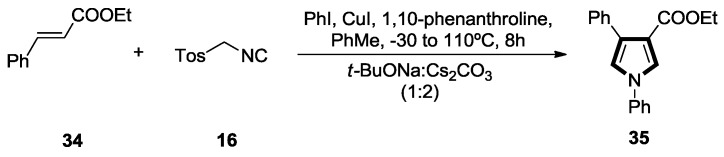
Synthetic route of *N*-arylated 3,4-disubstituted pyrrole **35**.

Hu’s group developed a procedure for the preparation of *N*-arylated 3,4-disubstituted pyrroles **35** from alkenes in the same year. They found that these compounds can be obtained when a mixture of ethyl 3-phenylacrylate **34**, TosMIC **16**, PhI, CuI, and 1,10-phenanthroline in toluene was treated with 3.0 equivalents of base at −30 °C for 10 min and then the resultant mixture was refluxed until the intermediate was completely exhausted. When a (1:2) mixture of *t*-BuONa to Cs_2_CO_3_ was used as base, **35** was obtained as a single product. In this procedure, *t*-BuONa served as a base for Van Leusen pyrrole synthesis and Cs_2_CO_3_ for the *N*-arylation, respectively (Scheme 9) [29].

**Scheme 10:**
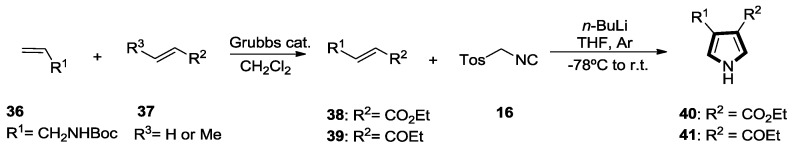
Synthesis of 3,4-disubstituted pyrrole compounds **40** and **41**.

In 2009, Poulard et al. designed a synthetic route for 3,4-disubstituted pyrrole compounds **40**, in which the 1,2-disubstituted Michael acceptors **38** are prepared by cross-methylation with compounds **36** and **37** that were used to react with TosMIC **16** to obtain **40**. Under the same conditions, compounds **41** can be also obtained when the R^2^ group was the ketone carbonyl-substituted Michael acceptor **39** (Scheme 10) [30]. 

**Scheme 11:**
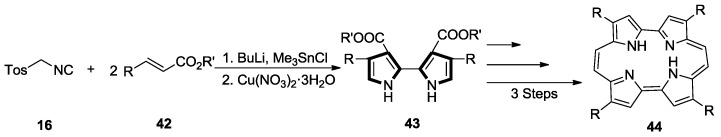
Synthesis of 2,2’-bipyrrole compounds **43**.

In the same year, the Sánchez-García group reported that 2,2′-bipyrroles compounds **43** were synthesized through the reaction of enesters **42** and TosMIC **16**. 2,7,12,17-tetraarylporphycenes **44** can be synthesized by using compounds **43** as raw materials. Porphycenes are of great value in the chemical industry and in biomedicine. During this reaction, there will also be a monopyrrole product formed, but if post-treated with dilute ammonia, the bipyrrole compound can be precipitated in ethyl acetate (Scheme 11) [31].

**Scheme 12:**
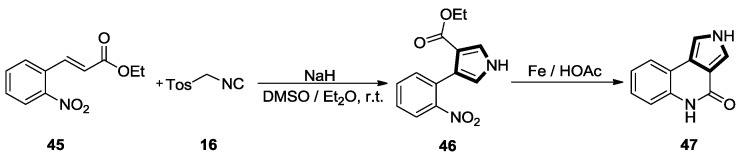
Synthetic route of pyrrole derivative **47**.

In 2012, Di Santo et al. described the formation of a pyrrole derivative **46** from (*E*)-ethyl-3-(2-nitrophenyl)acrylate **45** and TosMIC **16** under basic conditions. Then, nitro reduction and intramolecular cyclization into a lactam were further performed to synthesize 2*H*-pyrrolo[3,4-*c*]quinoline compound **47** (Scheme 12) [32]. 

**Scheme 13:**
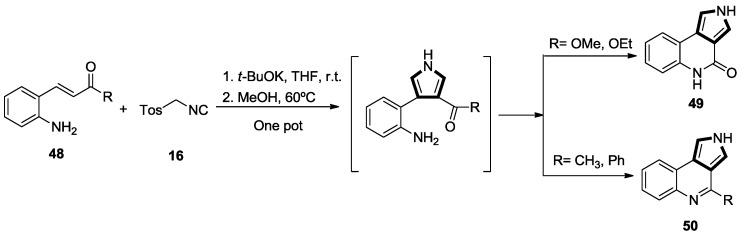
Synthesis of pyrrolo[3,4-*c*]quinolinone **49** and pyrrolo[3,4-*c*]quinolines **50**.

In 2014, the Ji group found that 2-aminoaryl acrylate **48** and TosMIC **16** undergo [3+2] cycloaddition under basic condition and could effectively synthesize pyrrolo[3,4-*c*]quinolinone **49** or pyrrolo[3,4-*c*]quinolines **50**. It is worth mentioning that the reacted pyrrole intermediate will both undergo an intramolecular condensation reaction of the ester or ketone with the amine during the formation of the quinoline ring. The reactions have good efficiency and practicality, and the yields can reach up to 73% (Scheme 13) [33]. 

**Scheme 14:**
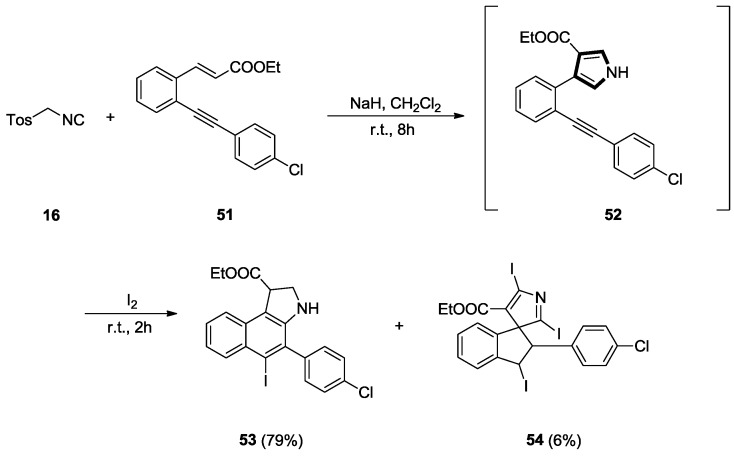
Synthesis of benz[*e*]indole derivative **53** and spiro[indene-1,3’-pyrrole] derivative **54**.

In 2015, our group developed an expedient and divergent tandem one-pot synthesis of benz[*e*]indole derivative **53** (79%) and spiro[indene-1,3’-pyrrole] derivative **54** (6%) from alkyne-tethered chalcones/cinnamates **51** and TosMIC **16**. The formation of intermediate **52** in this reaction involves a [3+2] cycloaddition with TosMIC **16** and electron-deficient ester **51** (Scheme 14) [34]. To our knowledge this reaction is the first example of intramolecular electrophilic cyclizations of alkynes with in-situ generated pyrroles.

**Scheme 15:**
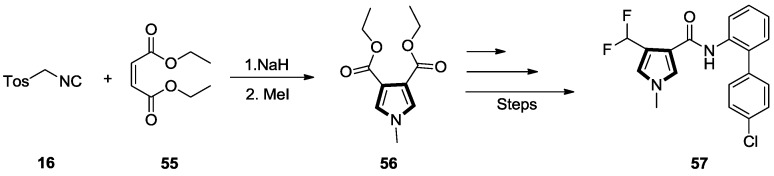
Synthesis of pyrrole carboxamide compound **57.**

In 2018, the Lamberth group reported a new synthesis route of pyrrolocarboxamide compounds **57**. They firstly used diethyl maleate **55** as starting material, which was converted with TosMIC **16** into the 3,4-dicarbethoxy-substituted pyrrole **56**. Then, after a series of reactions on the substituents, pyrrole carboxamide compound **57** was synthesized (Scheme 15) [35].

### 2.2. Alkenes with an Amide Group

**Scheme 16:**
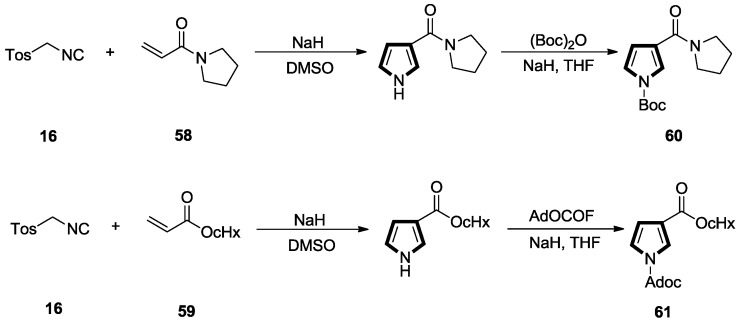
Synthesis of *N*-Boc pyrrole **60** or *N*-Adoc pyrrole **61**.

Donohoe and co-workers continued to expand the reaction to TosMIC **16** with acrylic acid pyrrolidide **58** to generate two pyrroles in reasonable yields in 1998. The compound was subsequently protected under standard conditions to yield the *N*-Boc pyrrole **60**. Under the similar conditions, *N*-Adoc pyrrole **61** could be obtained from cyclohexyl acrylate **59** and TosMIC **16**, respectively (Scheme 16) [36].

**Scheme 17:**
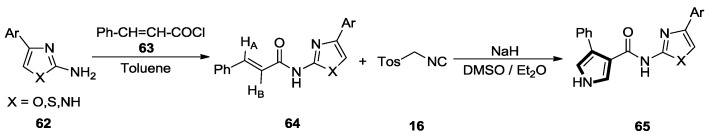
Synthesis of 4′-phenyl-*N*-(4-heteroaryl-2-yl)-1′*H*-pyrrole-3′-carboxamide compounds **65**.

In 2011, Padmavathi et al. condensed cinnamoyl chloride **63** with an aromatic heterocyclic compound **62** containing an amino group to prepare a series of aromatic heterocyclic cinnamic acid amide compounds **64**. Then, these electron-deficient alkenes were reacted with TosMIC **16** in the presence of NaH, DMSO and diethyl ether to prepare a series of 4′-phenyl-*N*-(4-heteroaryl-2-yl)-1′*H*-pyrrole-3′-carboxamide compounds **65**. According to a bioassay, compounds **65** have certain antibacterial activity against Gram-negative bacteria, and most of the compounds have the effect of inhibiting spore germination (Scheme 17) [37].

**Scheme 18:**
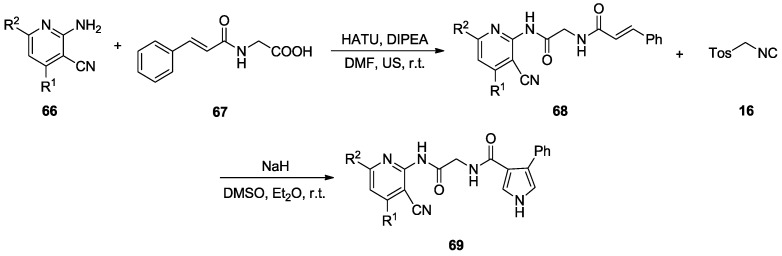
Synthesis of pyridinylcarbamoylmethyl pyrrolyl compounds **69**.

In 2017, Adivireddy et. al. developed a synthesis of pyridinylcarbamoylmethyl pyrrolyl compounds **69**. In this route, the synthetic intermediate (*E*)-*N*-((4-(aryl)-3-cyano-6-(aryl)pyridin-2-ylcarbamoyl)methyl)cinnamamide **68** was prepared by the condensation of 2-amino-4,6-diaryl-pyridine-3-carbonitrile **66** with 2-(cinnamamido)acetic acid **67** in the presence of *o*-(7-azabenzo-triazol-1-yl)-*N*,*N*,*N*,*N*-tetramethyluraniumhexafluorophosphate (HATU) and *N*,*N*-diisopropyl-ethylamine (DIPEA) in DMF under ultrasonication at room temperature. Then occurs the reaction of **68** with TosMIC **16** in the presence of NaH and in a solvent mixture of ether and dimethylsulfoxide (2:1) produced *N*-((4-(aryl)-3-cyano-6-(aryl) pyridin-2-ylcarbamoyl)methyl)-4-phenyl-1*H*-pyrrole-3-carboxamide **69**. The bis amido linked aromatized heterocycles pyrrolyl pyridines **69** exhibited excellent radical scavenging activity (Scheme 18) [38].

### 2.3. Alkenes with a Keto Group

**Scheme 19:**
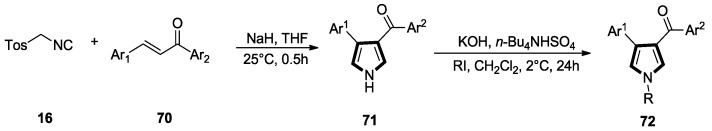
Synthesis of 3-aroyl-4-arylpyrroles **71** and *N*-substituted aryl-aroyl-pyrroles **72**.

In 2000, Dannhardt and co-workers reported that 1,3-diarylprop-2-en-1-ones **70** and TosMIC **16** were dissolved in THF as solvent to produce 3-aroyl-4-arylpyrroles **71** in the presence of NaH at room temperature for 0.5 h. Then compounds **71** were alkylated at the pyrrole nitrogen to afford an *N*-substituted aryl-aroyl-pyrroles **72** (Scheme 19) [39].

**Scheme 20:**
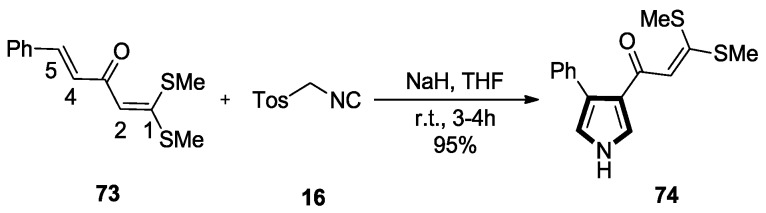
Synthesis of 3, 3-bis(methylthio)-1-(4-aryl-1*H*-pyrrol-3-yl)prop-2-en-1-one compound **74**.

The [3+2] cycloaddition of TosMIC with unsymmetrically substituted divinyl ketones occurs selectively on the less polar double bond. In 2007, the Rao group found that TosMIC **16** and cinnamoylketene dithioacetal **73** can selectively synthesize 3,3-bis(methylthio)-1-(4-aryl-1*H*-pyrrol-3-yl)prop-2-en-1-one **74** via [3+2] cycloaddition. This reaction occurs selectively on the less polar 4-position ene bond (Scheme 20) [40].

**Scheme 21:**
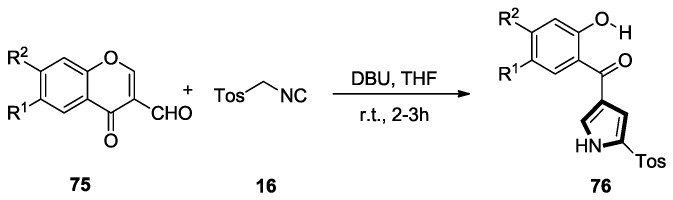
Synthesis of 2-tosyl-4-(2-hydroxybenzoyl)pyrroles **76**.

In the same year, Terzidis et al. reported that chromone-3-carboxaldehydes **75** were allowed to react with equimolar amounts of TosMIC **16** in the presence of DBU, in the aprotic nonpolar solvent THF at room temperature. As a result 2-tosyl-4-(2-hydroxybenzoyl)pyrroles **76** were isolated in good yield (Scheme 21) [41].

**Scheme 22:**
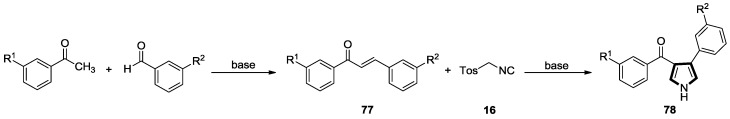
Synthetic route of pyrrole derivatives **78**.

In 2009, the Pérez group synthetized a series of pyrrole derivatives **78** by using TosMIC **16** with α,β-unsaturated carbonylic compounds **77**, which were obtained through Claisen-Schmidt condensation from the respective acetophenones and benzaldehydes substituted in *m*-position (Scheme 22) [42].

**Scheme 23:**
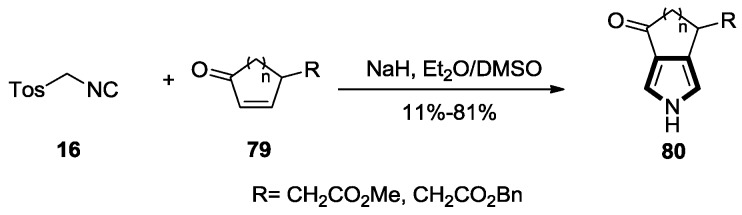
Synthesis of 3,4-fused cycloalkanopyrroles **80**.

In 2012, Kelly et al. found that the cyclic α,β-unsaturated ketone **79** can be used as a Michael acceptor for 1,3-dipolar cycloaddition to form pyrrole. The 3,4-fused cycloalkanopyrroles **80** were synthesized by reaction of **79** with TosMIC **16** and sodium hydride in a 3:1 solution of diethyl ether and dimethyl sulfoxide at room temperature, but the yield of the cyclization appeared to depend on the base-sensitivity of the Michael acceptor (Scheme 23) [43].

**Scheme 24:**
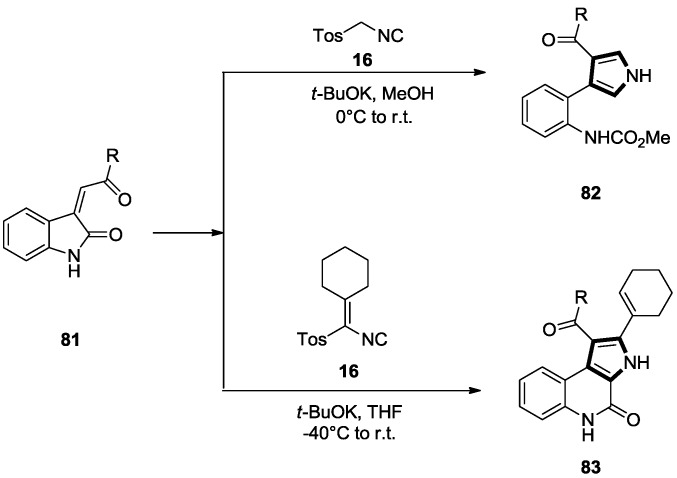
Synthesis of disubstituted pyrroles **80** and 3*H*-pyrrolo[2,3-*c*]quinolin-4(5*H*)-one derivatives **83**.

Similar reactions can occur with TosMIC derivatives. In 2013, the Ji group reported the reaction of TosMIC **16** with (*Z*)-3-(2-oxo-2-ethylidene)indolin-2-one derivatives **81** to give pyrrole derivatives **82**, and developed a simple and convenient synthetic approach to access of 3*H*-pyrrolo[2,3-*c*]quinolin-4(5*H*)-one derivatives **83** by the reaction of **81** with functionalized TosMIC derivatives **16** under basic conditions (Scheme 24) [44].

**Scheme 25:**
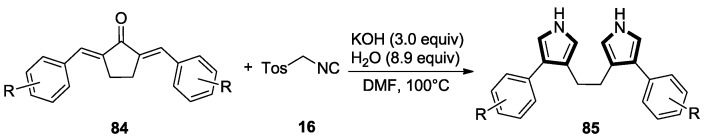
Synthesis of bridged 3,3′-dipyrrole derivatives **85.**

In the following year, Ji et al. also discovered a method for synthesizing bridged 3,3′-dipyrrole derivatives **85** by the reaction of dienone derivatives **84** with TosMIC **16**. In addition to the classical [3+2] cycloaddition reaction, this reaction also involves C-C bond cleavage caused by traces of water in the system. They also captured a spirocyclic intermediate, which is providing a new idea for the study of the construction of bispirocyclic compounds by isonitrile (Scheme 25) [45].

**Scheme 26:**
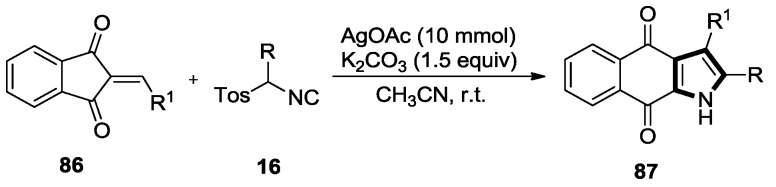
Synthesis of benzo[*f*]indole-4,9-diones compounds **87**.

In 2016, our group described a silver-catalyzed tandem reaction of TosMIC derivatives **16** with 2-methyleneindene-1,3-diones **86** to produce benzo[*f*]indole-4,9-diones compounds **87**. The reaction undergoes a domino [3+2]-cycloaddition/imidoyl anion cyclization/ring opening of cyclo-propanolate/aromatization and three C-C bonds are formed successively. The pyrrole ring is constructed while expanding a carbon atom to the original carbon ring (Scheme 26) [46].

**Scheme 27:**
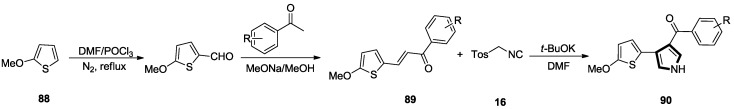
Synthesis of 4-substituted thienyl pyrrole compounds **90.**

In 2017, Mao and co-workers reported a synthesis of 4-substituted thienyl pyrrole compounds **90** via Vilsmeier-Haack formylation, aldol condensation and Van Leusen pyrrole synthesis using 2-methoxythiophene **88** as starting material. Compounds **90** have good selectivity and inhibition to tumor cells (Scheme 27) [47].

**Scheme 28:**
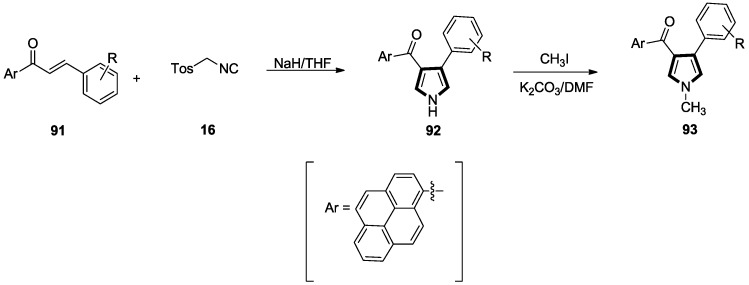
Synthesis of 3-aroyl-4-arylpyrrole compounds **93**.

In 2017, Shanmugam et al. described that chalcone **91** and TosMIC **16** undergo [3+2] cycloaddition under mild conditions to synthesize 3-aroyl-4-arylpyrrole compounds **92**. A new substituted carbamoylpyrrole **93** exhibiting moderate antibacterial activity against gram-positive bacteria and gram-negative bacteria was then synthesized by a substitution reaction with methyl iodide (Scheme 28) [48].

**Scheme 29:**
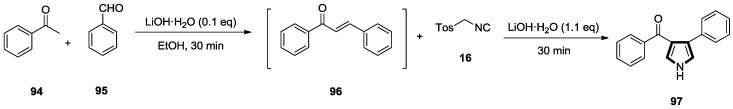
Synthesis of phenyl(4-phenyl-1*H*-pyrrol-3-yl)methanone **97**.

Solvation or dynamic solvent effects can affect the rate of [3+2] cycloaddition reaction between TosMIC and aromatic ketones. In 2013, Nair’s group discovered a lithium hydroxide mediated 3,4-disubstituted pyrrole synthesis method [49]. As shown in Scheme 29, acetophenone **94** was reacted with benzaldehyde **95** in the presence of LiOH·H_2_O in ethanol medium to afford 1,3-diphenylprop-2-enone **96** by an aldol condensation between the enolate and the electrophile. Then, TosMIC **16** and an additional equivalent of LiOH·H_2_O were added to the same system. As the reaction progressed, a white solid precipitated from the reaction medium, the product obtained was filtered, washed with water and ethanol, and characterized as phenyl(4-phenyl-1*H*-pyrrol-3-yl)methanone **97**. In this reaction system, due to the small size of Li^+^ ion, LiOH·H_2_O has obvious covalent character, which slows down the release of OH^-^. At the same time, a solvation effect occurs in the polar solvent to increase the yield.

**Scheme 30:**
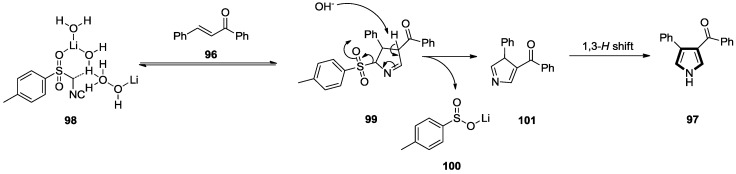
A plausible mechanism for the [3+2] cycloaddition reaction for TosMIC with LiOH·H_2_O.

A plausible mechanism for the reaction is depicted in Scheme 30. LiOH·H_2_O abstracts a proton from the methylene group of TosMIC to generate a carbanion which can be stabilized by the sulfonyl group. The intermediate 1,3-dipole undergoes [3+2] cycloaddition reaction to provide the cycloadduct **100**. Elimination of lithium *p*-toluenesulfinate under the action of a base to produce a C-3 substituted pyrrole derivative **101**. Finally, 1,3-hydride shift occurs to afford the product **97**. The *β*-substituent of the electrophile ends up at the C-4 position of the pyrrole ring.

**Scheme 31:**
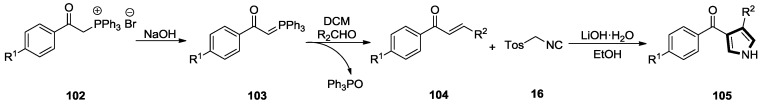
Synthesis of aroyl-substituted pyrroles **105**.

In 2016, Nair and co-workers also reported an aroyl-substituted pyrroles **105** synthetic route from phosphonium salt **102** as starting material. The phosphonium salt was neutralized with aqueous NaOH and extracted with dichloromethane to afford 1-phenyl-2-(triphenyl-phosphoranylidene)ethenone **103**. Further the compound was reacted with isobutyraldehyde in dichloromethane to generate the corresponding α,β-unsaturated ketone **104**. Upon completion of the reaction, dichloromethane was evaporated and the reaction mixture was triturated with hexane to remove triphenylphosphine oxide. Moreover, they found that LiOH·H_2_O gave good yields of the desired product as compared to NaOH and KOH. This might be due to better coordination power of lithium (Scheme 31) [50].

**Scheme 32:**
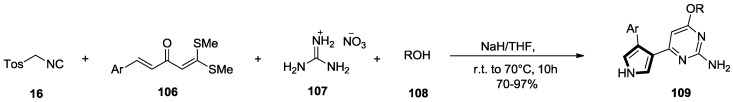
Synthesis of 6-pyrrolylpyrimidines **109.**

Multi-component Van Leusen pyrrole synthesis can also occur in alkenes with aromatic ketones. In 2014, the Shanmugam research group found that cinnamoylketene dithioacetal **106** undergo multi-component cycloaddition with TosMIC **16**, guanidine nitrate **107** and alkyl alcohol **108** in the presence of NaH/THF to furnish the target 6-pyrrolylpyrimidines **109** in excellent yields of 70–97% (Scheme 32) [51]. Because of the electron donating characteristics of the two methyl sulfanyl groups and the structural features of α,β-unsaturated carbonyl group, the arylvinyl double bond is more polarized than the ketene acetal double bond, which causes TosMIC **16** to selectively react at the arylvinyl double bond.

### 2.4. Alkenes with a Nitro Group

**Scheme 33:**
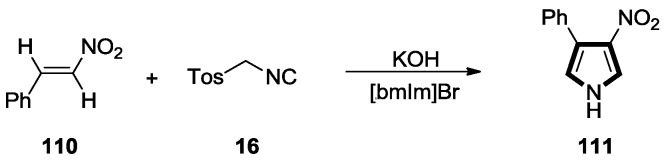
Synthesis of 4(3)-substituted 3(4)-niropyrrole **111**.

In 2009, Xiaoqi Yu and co-workers reported that 4(3)-substituted 3(4)-nitro-1*H*-pyrrole **111** can be synthesized from nitroene **110** and TosMIC **16** in the presence of the ionic liquid 1-butyl-3-methylimidazolium bromide ([bmIm]Br). This reaction can be widely applied to aromatic, aliphatic or heterocyclic substituted nitroolefins, and the recovered ionic liquid can be repeatedly used as a solvent without significantly reducing the yield (Scheme 33) [52].

### 2.5. Alkenes with a Cyano Group

**Scheme 34:**
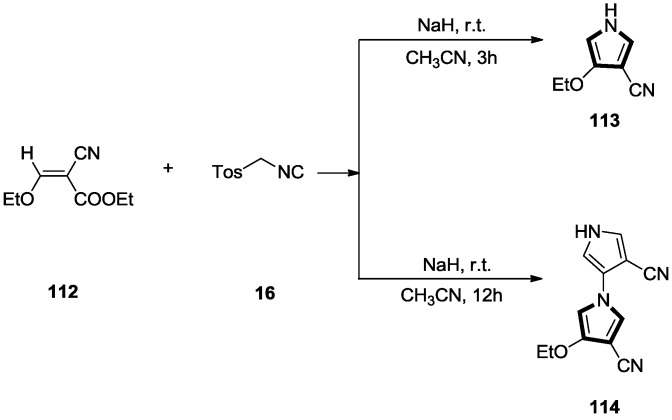
Synthesis route of 3,4-disubstituted pyrrole derivatives **113** and 1,3’-bipyrrole compound **114**.

In 2012, the Yongping Yu group reported that two equivalents of a cyano-substituted trisubstituted alkene **112** and TosMIC **16** were dissolved in anhydrous acetonitrile as a solvent and reacted in the presence of NaH at room temperature for 3 h to form disubstituted pyrrole derivatives **113**. And the 1,3’-bipyrrole **114** is obtained if the reaction time is extended to 12 h (Scheme 34) [53,54]. In the experiments, researchers also found that when keto and ester groups are simultaneously present in alkenes, due to the higher reactivity, keto group can be eliminated more easily than ester groups alone. In addition, group with larger steric hindrance can reduce the reactivity when they are present on alkenes.

### 2.6. Alkenes with an Aryl Group

**Scheme 35:**
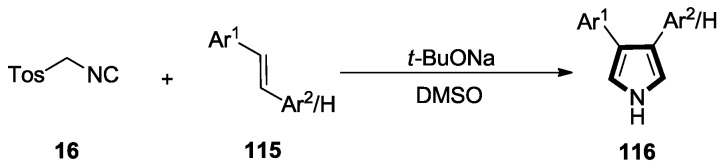
Synthesis of 3-aryl and 3,4-diaryl-(1*H*)-pyrroles **116**.

In 2002, Smith and co-workers developed a method for the one-step synthesis of 3-aryl and 3,4-diarylpyrroles **116** with good yields by readily available aryl or diaryl alkenes **115** with TosMIC **16** (Scheme 35) [55]. They found that the stronger the electron-withdrawing ability of the aryl group attached to the alkene in the substrate, the lower the temperature required for the reaction, the shorter the reaction time, and the higher yield. At the same time, the steric hindrance of the aryl group will act as a deterrent to the reaction. This phenomenon is particularly evident when the aryl group is ortho-substituted.

### 2.7. Other Alkene Synthons

**Scheme 36:**
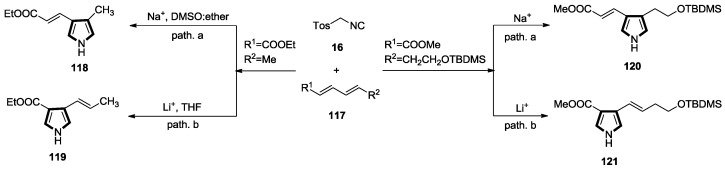
The effect of metal ion on the conjugate addition of TosMIC **16** anions to ethyl sorbate **117**.

Magnus et al. reported the effect of metal ion on the conjugate addition of TosMIC **16** anions to ethyl sorbate **117** in 1987. The sodium salt of TosMIC **16** in DMSO:ether reacted with **117** to afford pyrrole **118** (pathway a) in 80% yield, whereas the lithium salt of TosMIC **16** in THF afforded the addition adduct **119** in 61% yield (pathway b) (Scheme 36, left) [56]. On the basis of the research, Ganem and co-workers reported that condensation of **117** with the sodium anion of TosMIC in DMSO: ether gave regioisomers **120** in 1997. While using LiN(TMS)_2_ in THF at −78 °C, pyrrole derivate **121** was obtained as the only detectable product. The combination of more polar solvent and more highly dissociated anion favored reaction at the terminally polarized *δ*-position of dienoate **117**, leading to the desired 1,6-addition product (Scheme 36, right) [57].

**Scheme 37:**
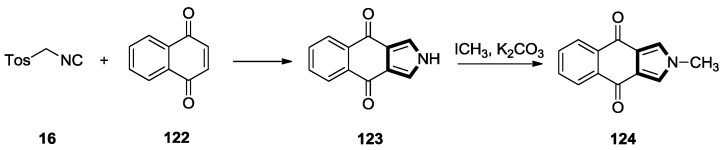
Synthesis of 2-methyl-2*H*-benz[*f*]isoindole-4,9-dione **124**.

Quinone can be also used for the Van Leusen pyrrole synthesis. In 1996, Di Santo et al. reported a synthesis of 2*H*-benz[*f*]isoindole-4,9-dione **123** from TosMIC **16** that reacts with Michael acceptor 1,4-naphthoquinone **122**. *N*-Methylation of the latter compound with iodomethane in the presence of anhydrous potassium carbonate afforded 2-methyl-2*H*-benz[*f*]isoindole-4,9-dione **124** (Scheme 37) [58].

**Scheme 38:**
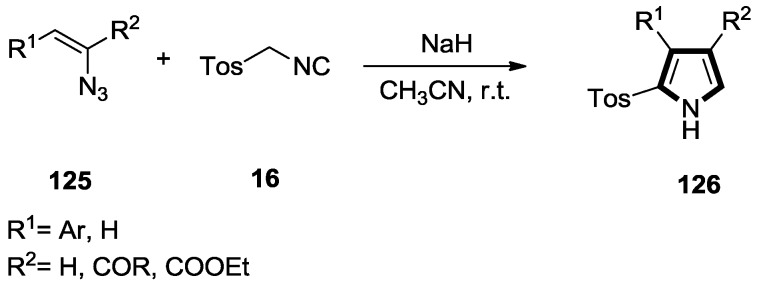
Synthesis of polysubstituted pyrroles **126**.

**Scheme 39:**
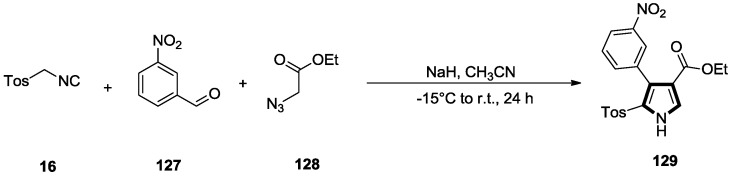
Synthesis of 2,3,4-trisubstituted pyrrole **129**.

In 2012, Yongping Yu and co-workers reported synthesis of polysubstituted pyrroles **126** from TosMIC **16** and vinyl azides **125** under mild conditions in the presence of base (Scheme 38) [59]. Additionally, they developed a Van Leusen three-component reaction as a synthesis of 2,3,4-trisubstituted pyrrole **129**, where a mixture of 3-nitrobenzaldehyde **127** and ethyl 2-azidoacetate **128** was stirred under the Knoevenagel condensation conditions using NaH as the base for 2 h at −15 °C, followed by addition of TosMIC **16**. The reaction mixture was then stirred at room temperature for 24 h to give the desired product **129** (Scheme 39) [59].

**Scheme 40:**
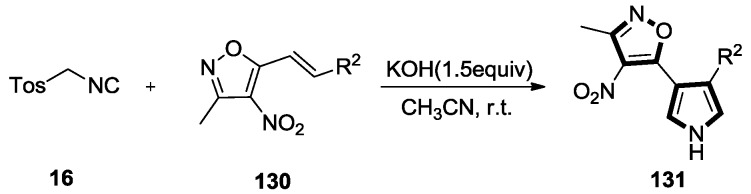
Synthesis of 3-isoxazole disubstituted pyrroles derivatives **131**.

**Scheme 41:**
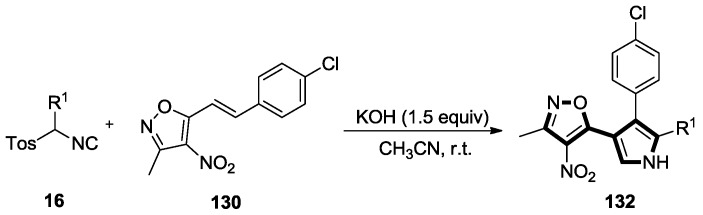
Synthesis of 3-isoxazole trisubstituted pyrrole derivatives **132**.

In 2017, our research group discovered that TosMIC **16** can undergo a [3+2] cycloaddition reaction with a styrylisoxazole compounds **130** to construct a series of 3-isoxazole disubstituted pyrrole derivatives **131** (Scheme 40) [60]. Under the same optimized reaction condition, the synthesis of the 3-isoxazole trisubstituted pyrrole derivatives **132** was achieved by using the TosMIC derivatives **16** (Scheme 41) [60]. This transformation is operationally simple, high-yielding, and displays broad substrate scope.

## 3. Synthesis of Pyrrole Derivatives by [3+2] Cycloaddition of TosMICs with Alkynes

**Scheme 42:**
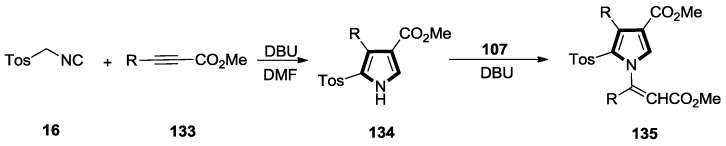
Synthesis of 2,3,4-trisubstituted pyrrole derivatives **134** and 1,2,3,4-tetrasubstituted pyrrole derivatives **135**.

Similar to alkenes, alkynes can also undergo [3+2] cycloaddition with TosMIC to synthesize pyrrole derivatives. As early as in 1979, Saikachi and co-workers found that acetylene ester **133** (2 equiv each) and TosMIC **16** can produce 2,3,4-trisubstituted pyrrole compounds **134** in the presence of DBU. The 1,2,3,4-tetrasubstituted pyrrole compounds **135** can be synthesized by another one-step addition reaction (Scheme 42) [61].

**Scheme 43:**
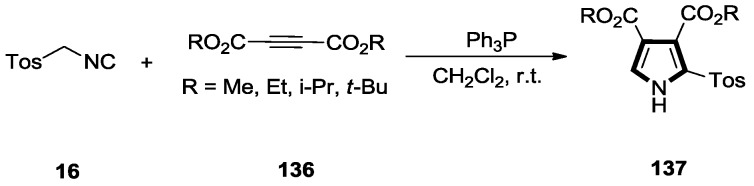
Synthesis of 2,3,4-trisubstituted pyrrole derivatives **137**.

In 2006, Alizadeh et al. found that dialkyl acetylenedicarboxylates **136** react with TosMIC **16** in the presence of Ph_3_P to form dialkyl 2-[(4-methylphenyl)sulfonyl]-1*H*-pyrrole-3,4-dicarboxylates **137**. In this reaction, the nucleophilic addition of Ph_3_P to the acetylenic esters further increases the reactivity of the substrate as an electron-withdrawing group (Scheme 43) [62].

**Scheme 44:**
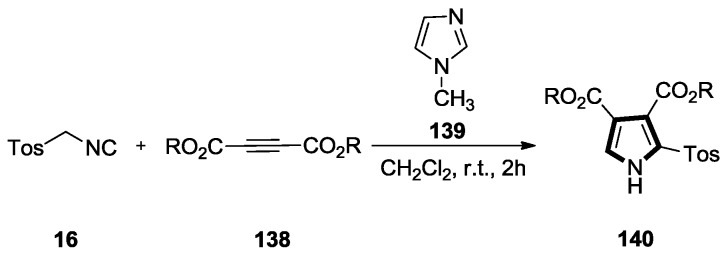
Synthesis of 2,3,4-trisubstituted pyrrole derivatives **140**.

In 2011, the Adib group developed a protocol that is different with respect to the common shortcomings such as long reaction time, low yield, expensive raw materials, and harsh reaction conditions. A mixture of TosMIC **16**, and a dialkyl acetylenedicarboxylate **138**, in the presence of a catalytic amount of 1-methylimidazole **139** undergoes a smooth addition reaction in anhydrous CH_2_Cl_2_ at room temperature to afford 2,3,4-trisubstituted pyrroles **140** in yields of 90–95% (Scheme 44) [63]. 

## 4. Conclusions

In summary, the Van Leusen [3+2] cycloaddition reaction based on TosMICs and electron-deficient compounds is involved in the construction of pyrrole and its derivatives because of its advantages such as simple and convenient synthesis substrate, diverse products, etc., and will play an increasingly important role in the synthesis of bioactive pyrrole derivatives in the pharmaceutical synthesis. In recent years, some research progress has been made, which provides new ideas for the synthesis of polysubstituted pyrrole ring framework. In the future, there will be a focus on developing more types and higher selectivity 2-carbon synthons in subsequent research to increase the expansion of the reaction.

## Figures and Tables

**Table 1:** Some typical pyrrole derivative chemical structures and the physiological functions.

Physiological Functions	Chemical Structures		References
Antibacterial	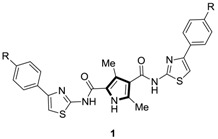	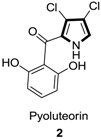	[1,2]
Antifungal	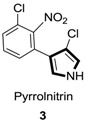	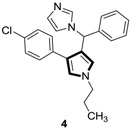	[3,4]
Anti-inflammatory	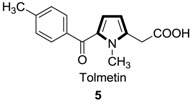	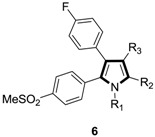	[5,6]
Antiviral	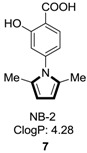	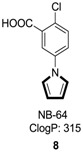	[7]
Antimalarial	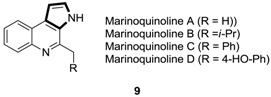		[8]
Anticancer	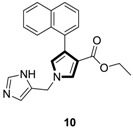	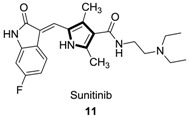	[9,10]
Antiparasitic	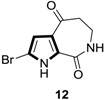	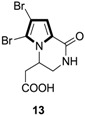	[11]
Enzyme inhibitor	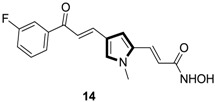	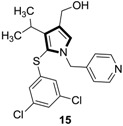	[12,13]
